# Dementia, Depression, and Associated Brain Inflammatory Mechanisms after Spinal Cord Injury

**DOI:** 10.3390/cells9061420

**Published:** 2020-06-08

**Authors:** Yun Li, Tuoxin Cao, Rodney M. Ritzel, Junyun He, Alan I. Faden, Junfang Wu

**Affiliations:** 1 Department of Anesthesiology and Center for Shock, Trauma and Anesthesiology Research (STAR), University of Maryland School of Medicine, Baltimore, MD 21201, USA; yun.li@som.umaryland.edu (Y.L.); tcao@som.umaryland.edu (T.C.); rritzel@som.umaryland.edu (R.M.R.); jhe@som.umaryland.edu (J.H.); afaden@som.umaryland.edu (A.I.F.); 2 University of Maryland Center to Advance Chronic Pain Research, University of Maryland, Baltimore, MD 21201, USA

**Keywords:** spinal cord injury, depression, cognition, dementia, neuroinflammation, brain

## Abstract

Evaluation of the chronic effects of spinal cord injury (SCI) has long focused on sensorimotor deficits, neuropathic pain, bladder/bowel dysfunction, loss of sexual function, and emotional distress. Although not well appreciated clinically, SCI can cause cognitive impairment including deficits in learning and memory, executive function, attention, and processing speed; it also commonly leads to depression. Recent large-scale longitudinal population-based studies indicate that patients with isolated SCI (without concurrent brain injury) are at a high risk of dementia associated with substantial cognitive impairments. Yet, little basic research has addressed potential mechanisms for cognitive impairment and depression after injury. In addition to contributing to disability in their own right, these changes can adversely affect rehabilitation and recovery and reduce quality of life. Here, we review clinical and experimental work on the complex and varied responses in the brain following SCI. We also discuss potential mechanisms responsible for these less well-examined, important SCI consequences. In addition, we outline the existing and developing therapeutic options aimed at reducing SCI-induced brain neuroinflammation and post-injury cognitive and emotional impairments.

## 1. Introduction

As the primary relay center of neural transmission between the brain and the rest of the body, damage to the spinal cord can be a devastating event. Chronic evaluation of spinal cord injury (SCI) has focused on sensorimotor deficits, neuropathic pain, bladder/bowel dysfunction, loss of sexual function, and emotional distress [1,2,3,4,5]. Although clinical studies have reported that 40–60% of SCI patients show cognitive and emotional deficits [6,7,8,9,10,11,12,13], the cause of such changes has been debated because of potentially confounding factors such as concurrent traumatic brain injury (TBI). However, studies addressing this issue in SCI cases without TBI confirmed impairments in cognitive function [7,8,9,11,14,15]. Such cognitive/emotional impairments can compromise not only quality of life but also rehabilitation and recovery.

Prior research on physiopathology following SCI has focused on injured spinal cord and its anterograde and retrograde pathways to the brain. Conversely, limited work has examined potential effects of SCI on brain regions that impacts learning and memory or emotions. Identifying mechanisms responsible for these less-well-examined, important SCI consequences could provide targets for more effective therapeutic interventions that improve outcome. To date, our mechanistic understanding of this disease process is informed by clinical studies but requires deeper exploration using well-controlled experimental models. In this review, discuss the recent work on cognitive disorders following SCI and also attempt to highlight potential mechanisms that may explain how biochemical, physiological or behavioral changes induced by SCI affect brain circuitry. In addition, we address potential therapeutic options, with emphasis on reducing SCI-induced brain neuroinflammation for limitation of post-injury cognitive and emotional impairments.

## 2. Neuropsychological Abnormalities and Pathophysiological Alterations in the Brain in Patients with SCI

The current high volume of clinical evidence indicates substantial cognitive impairment in individuals with SCI and no association with level of injury [13]. Roth et al. reported that SCI patients can show decreases in attention span, concentration, memory function, and learning [11], suggesting a wide range of cognitive disabilities. These reported changes in psychological state may not be that surprising given the limited range of mobility that compromises active participation in social, occupational, physical, and leisure activities. A nationwide population-based cohort study in Taiwan found that SCI patients have a significantly higher risk for developing dementia, providing the epidemiologic evidence that SCI can contribute to cognitive decline [14]. Overall, SCI patients are 13 times more likely to develop cognitive impairments [16]. As SCI patients may have concurrent brain injury, some have questioned whether isolated SCI can itself cause cognitive decline and depression [17]. However, there is a high prevalence of major depression in both traumatic and non-traumatic SCI patients, which suggests that depression reflects physiological changes following SCI rather than concurrent traumatic brain injury [18]. Citing these epidemiological studies, the high prevalence of depression and dementia are increasingly recognized as serious secondary complications that can impede recovery of patients.

Mood disturbances are also common in SCI patients. Although the statistics differ across studies, the prevalence of depression, anxiety, and posttraumatic stress disorder (PTSD) are consistently increased in patients with chronic SCI [19,20]. In a study of chronic SCI patients in Australia, 46% reported symptoms indicative of mood disturbance [21]. Another study found that 63% of SCI patients experienced depression within 3 months of injury [22]. One recent study confirmed the high incidence of depression after SCI [23], but with notable sex differences (53.6% of women versus 25% of men); this striking gender variation requires confirmation. Follow-up studies showed that these disorders persisted throughout their lifetime, and intervention was only able to resolve a third of the patients’ mood disorders [24,25].

The concept that SCI causes remote pathophysiological changes in the brain has largely been restricted to localized brain regions associated with sensorimotor modulation pathways. Magnetic resonance imaging (MRI) studies showed that SCI causes extensive long-term reorganization of the cerebral cortex [26,27,28], and complete thoracic SCI patients have decreased grey matter volume in primary motor cortex that is consistent with neuronal loss and/or atrophy [29]. Functional MRI confirmed axonal degeneration and demyelination in the cortex after cervical SCI [30]. Furthermore, prospective longitudinal functional MRI studies reported that SCI can cause progressive reduction in grey matter volume not only in the sensorimotor cortex but also in the regions not directly connected to the injury site; the latter include the cerebellar cortex, medial pre-frontal cortex, and anterior cingulate regions that are critical for the processing of emotional relevant information or modulation of attentional states [29,31,32,33,34]. Perhaps consistent with these observations, SCI has been found to cause impairments in distributed cortical/subcortical networks that are engaged in information processing functions [9]. Moreover, myelin-sensitive MRI parameters measured at one year after SCI were reduced not only within, but also beyond, the atrophic lesion area [31]. Progressive ventricles enlargement and cerebrospinal fluid volume increases provide additional information on the neurodegenerative processes in the brain after SCI [35]. Collectively, these findings support the hypothesis that widespread profound alterations to the brain occur after SCI, which may contribute to the development of cognitive and mood disorders.

## 3. Experimental Evidence of SCI-Mediated Impairment of Cognition and Depression as Well as Brain Pathology

Experimental models of SCI show that rodents develop cognitive and mood disabilities that parallel those seen in human patients. The first experimental observation potentially suggesting a connection between SCI and worsening brain function used projectile injury to the thorax of pigs wearing body armor [36]. The authors found significant mitochondrial swelling and vacuolated cytoplasm in the hippocampus of the injured group, concurrent with learning and memory impairment. However, due the nature of the insult, the changes reported in this study may not be exclusive to the spinal cord—rather, the transmission of impact force caused by the body armor may have directly affected the brain. Observation of depression-like behavior in contusion SCI was noted by Luedtke et al., who showed abnormalities using a tail suspension test for depressive-like behavior, which could be reversed by administering the anti-depressant drug fluoxetine [37]. Studies from our laboratory [38,39,40] demonstrated that male mice and rats subjected to lower thoracic contusion injury suffered not only from long-term deficits in locomotor function but also gradual loss of cognitive abilities as late as eight weeks after injury. We implemented a battery of behavioral tasks designed to minimize the confounding effects of motor deficits on cognitive performance, including non-spatial recognition memory evaluated by a novel object recognition (NOR) test and hippocampus-dependent spatial working memory examined by the Y maze testing [40]. We found that SCI caused significant impairment in spatial and non-spatial learning, in addition to loss of memory retention. Subsequent work from our group demonstrated that SCI in male mice display increased anxiety-like behavior in an open field test, as well as increased anhedonia in a sucrose preference test, both indicative of anxiety/depression-like behavior [40]. These findings highlight the potential utility of preclinical modeling to try to elucidate key mechanisms and pathology of SCI-induced behavioral alterations.

**Figure 1:**
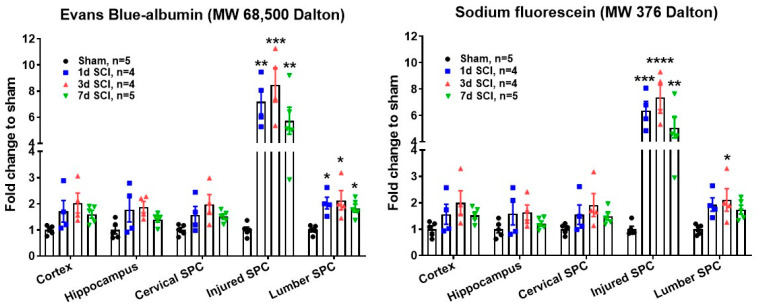
Blood–brain barrier (BBB) and blood–spinal cord barrier (BSB) permeability assay. A T10 spinal cord contusion injury (moderate/severe injury) was produced in young adult C57BL/6 male mice (2–3 months old) using the Infinite Horizon spinal cord impactor as previously described [38,40]. 100 μL of saline solution containing 10% sodium fluorescein and 2% Evans blue was injected by tail vein (100 μL/mouse) at 1 d, 3 d, and 7 d after SCI. At 30 min after dye injection, mice were perfused with 100 mL of saline and injured thoracic spinal cord (SPC), lumbar and cervical SPC, as well as cerebral cortex and hippocampus were dissected for fluorescent assay (sodium fluorescein at 485/528 nm, Evans blue at 470/680 nm). * *p* < 0.05, ** *p* < 0.01, *** *p* < 0.001, **** *p* < 0.0001 vs. Sham. One-way ANOVA following Tukey’s multiple comparisons test.

Following SCI, the blood–spinal cord barrier (BSCB) is disrupted, which is detrimental to tissue recovery [41,42,43]. Yet, whether the blood–brain barrier (BBB) is also compromised following SCI remains unclear. Evans blue, a small molecule that binds rapidly to albumin, provides a useful way to characterize the breakdown of endothelial barriers [44,45]. In addition, low molecular weight (MW) inert tracers like NaF has high sensitivity for determining the breakdown of microvessels in injured CNS [45]. Recently, we examined the permeability of not only the BSCB in injured thoracic spinal cord and lumbar tissue, but also the BBB in the brain after SCI in male mice using both Evans blue and NaF dyes. As indicated in Figure 1, SCI mice exhibited significantly increased permeability of BSCB at injured site beginning 24 h; this peaked at three days and remained elevation at seven days. BSCB permeability was also increased at lumbar regions up to seven-days post-injury. However, neither cervical spinal cord nor the brain sub-regions (cerebral cortex and hippocampus) showed increased BBB permeability.

However, consistent with the clinical pathology, increased activation and functional reorganization in the somatosensory cortex could be observed in the immediate aftermath of SCI [46,47]. Of the few studies that examine neuronal function in the brain after SCI, signs of neurodegeneration, mitochondrial swelling, and vacuolated cytoplasm were observed in the hippocampus along with elevated levels of injury biomarkers in the cerebral spinal fluid [36,48]. In addition, rodent models of experimental SCI also induce neuronal atrophy. Increased brain expression of Calbindin-D (28 K), caspase-3, and Bax protein are associated with increased neuronal apoptosis in the primary motor cortex [49,50,51]. Cell loss in this region was shown to reduce motor evoked potentials, indicating that SCI alters the excitability and functionality of upper motor neurons [49]. Interestingly, injecting/transplanting brain derived neurotrophic factor (BDNF)-secreting cells at the SCI lesion site ameliorated pyramidal neuron loss in the rhesus macaque, providing further mechanistic insight into SCI-induced brain injury, and suggesting that mitigating injury in the spinal cord via of supplementation of neurotrophic factors or otherwise may limit or even prevent neuronal damage in the brain [52]. However, additional examination is needed as reports from subsequent studies have been mixed, with some showing no observable neuronal loss in the cortex following SCI [53,54]. Reasons for these disparate results are not fully understood; however, injury severity, time after injury, and differences in experimental modeling can all affect pathological outcomes. Further investigation of the underlying mechanisms is needed to fully understand SCI-induced cognitive and mood disorders.

## 4. Neuroinflammation and Neurodegeneration in the Brain after SCI

**Figure 2:**
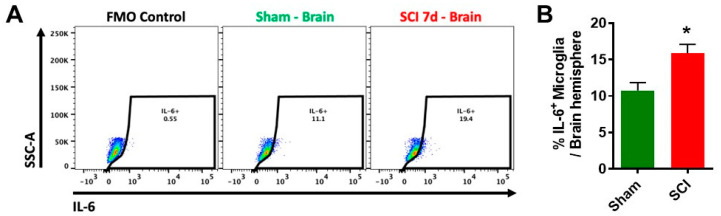
Increased proinflammatory cytokine IL6^+^ microglia occur in the brain after SCI. A T10 spinal cord contusion injury (moderate/severe injury) was produced in young adult C57BL/6 male mice (2–3 months old) using the Infinite Horizon spinal cord impactor as previously described [38,40]. At seven days after injury, mice were perfused with ice-cold PBS, and the brain hemisphere was isolated for preparation of single cell suspension using standard FACS protocol. Cells were then incubated with Fc Block prior to staining with primary antibody-conjugated fluorophores: CD45-Bv421, CD11b-APC/FireTM750, and Zombie AquaTM viability dye. Cells were then subject to fixation/permeabilization for cytokine labeling (i.e., IL-6-PE). All reagents were obtained from BioLegend Inc. (**A**) A representative histogram shows the relative frequency of IL-6-positive brain-resident microglia at seven days after sham and SCI surgery. FMO: fluorescence minus one; SSC-A: side scatter-area. (**B**) The percentage of IL6-positive brain microglia is quantified. N = 4 (Sham) and 5 (SCI) mice. * *p* < 0.05 vs. Sham with Mann–Whitney test.

Chronic inflammation occurs in pain regulatory areas such as brainstem and thalamus after SCI, with posttraumatic hyperesthesia associated with plasticity or electrophysiological alterations [55,56,57,58,59,60,61,62,63,64,65,66,67,68,69,70,71]. Chemokines CCL2 and CCL3 are chronically expressed in thalamus, hippocampal CA3 and dentate gyrus (DG), and periaqueductal gray matter after severe SCI [61]. Our recent autoradiography studies [39] in male rats after SCI, using a new translocator protein 18 kDa (TSPO) ligand [^125^I] IodoDPA-713 [72] (a new probe for imaging inflammation in clinical PET studies), revealed that cortex, thalamus, hippocampus, cerebellum, and caudate/putamen all showed chronic brain inflammation. Moreover, flow cytometry analysis demonstrated that moderate/severe SCI in C57BL/6 male mice caused significantly increased levels of proinflammatory cytokine IL6 in the brain (Figure 2). These data complemented microscopy findings showing chronic microglial activation in brain after SCI [38,39,40,73,74]. Glial activation was confirmed in the sub-granular zone and molecular layer of the DG in the hippocampus in a severity-dependent manner; such activation was only found in moderate and severe SCI, but not mild [73]. Moreover, increased levels of IL1α and TNFα were observed in the hippocampus of rats with anxiety/depressive-like behavior after SCI [75]. Modulating inflammation has recently been shown to improve mood in patients with SCI [76]. Thus, isolated thoracic SCI in rats and mice causes widespread progressive chronic neuroinflammation, leading to neurodegeneration in key brain regions associated with cognitive dysfunction and depression. However, the precise molecular mechanisms underlying these changes have not been elucidated.

Neurogenesis, the process of generating new neurons from neural stem cells, is usually restricted to fetal and peri-natal time periods in the developing brain and ends shortly after birth. In the adult brain, however, neurogenesis may still be observed in the subgranular zone of the DG in the hippocampal and in the subventricular zone in the lateral wall of the lateral ventricles [77]. Psychiatric and neurodegenerative disorders are often associated with altered neurogenesis [78,79], some theorize cognitive deficits in the elderly result from a gradual decrease in hippocampal neurogenesis during the aging process [80]. Impaired neurogenesis is also seen in the hippocampus with chronic stress and depression [81,82]. With regard to SCI, the data are less clear. Glial activation has been shown to reduce neurogenesis [83]. By extension, SCI-induced brain inflammation could also impact neurogenesis. Several studies reported decreased neurogenesis in the brain at chronic time periods after SCI [38,40,73,84]. Another study, however, found an increase of gliogenesis in the brain following SCI, whereas neurogenesis remained unaltered [85]. This could reflect differences in timing and location, as the former examined the effects of a cervical injury at 90 days after SCI, whereas the latter investigated effects of a thoracic injury at 42 days after SCI. Injury severity may also play a role. Indeed, Jure et al. reported that although severe and moderate SCI reduced neurogenesis, mild SCI did not [73]. To this end, our laboratory has shown that discrete regions of the brain exhibit significant signs of cell cycle arrest and decreased numbers of immature neurons in the male murine hippocampus after SCI to the T10 segment [38,40]. These findings, combined with the widely explored theory of impaired neurogenesis being one of the underlying mechanisms of cognitive decline, may provide an explanation as to why SCI patients have a significantly higher risk of dementia. Although adult neurogenesis is clear in rodents, whether and to what extent adult neurogenesis occurs in humans remains controversial [86,87].

## 5. The Influence of Aging on SCI-Mediated Cognitive Impairments

According to the report from the National Spinal Cord Injury Statistical Center (NSCISC), the average age at the time of injury has increased from 29 years in the 1970s to approximately 42 years in currently (https://www.sci-info-pages.com/facts.html). Moreover, today individuals with SCI have an average life expectancy of more than 30 years; however, given that such injuries now occur more frequently in older persons than previously, they are more susceptible to problems associated with ageing and dementia. Indeed, cognitive functioning is negatively correlated with age in individuals with SCI [13].

It is well known that ageing potentiates inflammation and neurodegeneration at the injury site, and impairs recovery from CNS trauma [88,89,90,91,92]. Aging is also an important pathogenic factor in other neurodegenerative disorders, including Alzheimer’s disease (AD) [93,94]. Recent large-scale longitudinal population-based studies [14,16,20] showed that patients with SCI are at higher risk of developing dementia than non-SCI patients, indicating that SCI is a potential risk factor for dementia. Therefore, it is intriguing to investigate the mechanisms of SCI-induced dementia or its relationship to age of onset or age-related neurodegenerative disorders such as AD. On the other hand, elderly patients with impaired cognitive function are at risk of sustaining falls [95,96], which are an important cause (30%) of SCI (NSCISC). Furthermore, patients with dementia such as AD, could have higher risk of falls [97,98,99], and therefore increased risk of SCI. Thus, there may be an emerging confluence of SCI and dementia in the elderly population that represents a significant unmet healthcare challenge. We and others [37,38,39,40,49,51,73,74,75] show that cognitive impairments and depression are detected weeks to months after isolated thoracic experimental SCI and that progressive neuronal loss and microglial activation occur in brain regions involved in memory and learning. Extending these observations, using aging and aged animals should help elucidate underlying mechanisms and generate new treatment approaches.

## 6. Potential Mechanisms for SCI-Mediated Brain Pathology

### 6.1. Anterograde and Retrograde Mechanisms

Although mounting evidence now indicates that SCI causes a significant change in brain function, the underlying anatomical pathways and molecular mechanisms are not clear. Anterograde and retrograde connections between the spinal cord and various brain regions certainly exist. The contusion site may also affect distal brain regions via the production and diffusion of chemokines. For instance, the chemokine cysteine-cysteine chemokine ligand 21 (CCL21) was shown to be produced in lumbar dorsal horn neurons around the SCI lesion site, but was also found in the thalamus, cerebral cortex, and hippocampus at later time points [39,40,55,100,101]. Subsequent work by De Jong et al. demonstrated that neuronal CCL21 is sorted into large dense-core vesicles and transported into axons, providing evidence for the directed transport from one neuron to another [100]. Indeed, it is now accepted that CCL21 is transported from/throughout neuronal processes into presynaptic structures [101]. This would suggest that chemokines can be transported in an anterograde manner to the areas distant from the lesion site. In addition to CCL21, SCI increases expression of CCL2 and its receptor CCR2 in the thalamus, hippocampus, and periaqueductal gray matter at chronic time points after injury [61]. Further examination is needed to determine the functional role of these and potentially other chemokines once they reach the brain via anterograde (e.g., through spinothalamic pathways) transport.

### 6.2. Distal Release of CCL21

**Figure 3:**
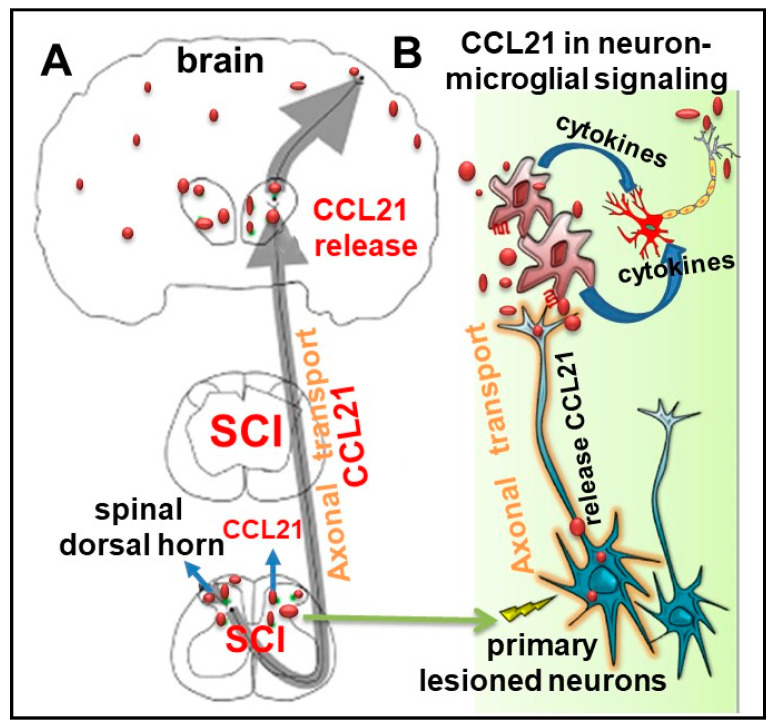
A schematic diagram for the cysteine-cysteine chemokine ligand 21 (CCL21) axonal transport and its role in neuron-microglia signaling following spinal cord injury (SCI). (**A**) CCL21 in damaged neurons is sorted into vesicles that are transported via axons and reach presynaptic structures and secreted into parenchyma. (**B**) Distal release of CCL21 triggers microglial activation at distant site from the lesion, which release proinflammatory cytokines and microparticles reinforcing neuronal damage. The latter causes more distant CCL21 release through their axonal transport.

Neuronal CCL21 is a potent microglial-activating chemokine [55,100,101,102,103,104]. We and others have reported [39,40,55,57] that SCI triggers up-regulation of CCL21 in a number of brain regions including thalamus, hippocampus, and cerebral cortex; increased thalamic CCL21 levels are associated with microglial activation and hyperpathia. CCL21 is not detected in healthy neurons, glia cells, or other non-neuronal cells in the brain [105]. That CCL21 is specifically expressed in injured neurons and that may act as a signal from damaged neurons to microglia were reported in 2001 [103]. Subsequent studies [100,101,104,106] confirm that CCL21 is synthesized by damaged neurons, transported by axons, and released to activate microglia-a phenomenon that has also been observed in humans [107,108]. Thus, CCL21 in the CNS is exclusively expressed in injured neurons and can cause neuroinflammation at sites distant from the injury site. In SCI [55], thalamic levels of CCL21 are rapidly reduced after spinal cord blockade using lidocaine, supporting the view that SCI elevates CCL21 levels in the brain. Increased CCL21 in more distant neurons after SCI [40,55,57] may reflect subsequent damage to second order neurons by the induced microglial activation. It is plausible that (1) activated microglia by CCL21 release pro-inflammatory cytokines that are toxic to surrounding neurons, leading to more distant CCL21 release through their axonal transport, consistent with the evidence for elevated CCL21 signals in broad brain regions at later time-points after SCI [40]; (2) such delayed increases of CCL21 in more distant regions are associated with progressive chronic neuroinflammation [39]. An alternate hypothesis is that activated microglia release microparticles, which contain pro-inflammatory molecules that can contribute to the spread of brain inflammation. It is known that microparticles are extracellular vesicles that play a critical role in cell-cell communication, including between immune cells and their targets. We recently reported [109] that microglial-derived microparticles can mediate progressive, spreading neuroinflammation after TBI. A schematic diagram for CCL21 axonal transport and its effects on microglial activation is illustrated in Figure 3.

Although CCL21 is up-regulated under pathological conditions, the responsible receptor for the CCL21-dependent microglial activation is unclear [105,110,111]. There are two known receptors for CCL21 in mice: CCR7 and CXCR3 [112]. CCR7 is not found in microglia under basal conditions, but it can be induced in vitro and in vivo [103,113,114,115]. In contrast, CXCR3 is constitutively expressed in cultured microglia and in acutely isolated microglia [114]. However, neither the deficiency of CCR7 nor CXCR3 had a major impact on development of neuropathic pain, in contrast to the striking phenotype in the absence of their ligand CCL21. In agreement with earlier studies [102,105,106,110], we were not able to detect CCR7 mRNA or CXCR3 mRNA in the brain, even after SCI. Thus, neither in vitro nor in vivo studies have clearly defined a functional role for CCL21 signaling in microglia. However, the underlying mechanisms of CCL21-triggered detrimental microglial activation and associated functional outcomes, including cognitive deficits are intriguing for future investigation.

### 6.3. Systemic Immune Functions

Another serious complication in SCI patients is systemic immune dysfunction. The peripheral immune response is complicated, with some studies reporting an increase in systemic inflammatory activation and others showing impaired immunological responses. SCI induces systemic increases in immune cells and pro-inflammatory factors [116]. Free radical production increases significantly in neutrophils isolated from SCI patients compared to control subjects [117]. Expression of the NADPH oxidase subunit gp91(phox) and nitric oxide synthase are increased in blood [117]. SCI increases the NOD-like receptor family, pyrin domain containing 3 (NLRP3) inflammasome formation, in peripheral tissues [118]. Pro-inflammatory cytokine levels of TNFα, IL-1β, and IL-6 are increased in serum after rat spinal cord ischemia injury model [119]. SCI can also disturb neuroendocrine functions, by activating the hypothalamic-pituitary-adrenal axis, causing systemic inflammation by inducing production of macrophage migration inhibitory factors from pituitary gland [120]. Collectively, increased free radical production, inflammasome activation, and cytokine production may exacerbate leukocyte infiltration in the brain and promote excessive microglial activation and chronic inflammation [116].

Contrary to systemic immune activation, SCI can also impair systemic immune function. In clinical studies, SCI survivors have increased morbidity for infections due to the development of SCI-induced immune depression syndrome (SCI-IDS), which is a system-wide deficit of immune surveillance [121]. SCI-IDS is thought to be caused by severing thoracolumbar spinal cord projections, disrupting sympathetic nervous system signaling [122]. Hallmarks of SCI-IDS include splenic atrophy, leukopenia, reduced anti-microbial activity, and impaired humoral immunity. Although SCI-IDS may have a positive effect by reducing the potential for auto-immune damage to the CNS [123], these complications compromise the patient’s immunity, resulting in higher cost of care and rehospitalization incidences. Importantly, pathological changes to the immune system as a whole may promote brain inflammation and significantly impede neurological recovery after SCI.

### 6.4. Chronic Neuropathic Pain

Chronic neuropathic pain, a common secondary complication of SCI, also plays a major role in the development of depression [18,124]. In the context of clinical cases, persistent pain has been reported in approximately 65% of people affected by SCI [3]. In a cohort study by Perry et al., approximately half of the individuals undergoing rehabilitation after SCI reported “moderate” or “severe” pain [125]. Persistent pain experienced by SCI patients is thought to exacerbate cognitive dysfunction and be detrimental to recovery. In fact, Murry et al. found that post-SCI pain significantly correlated with neurological behavior, and can be used as a predictor of cognitive function, emotional function and quality of life [15]. In a recent Swedish survey, neuropathic pain was one of the most critical factors contributing to the low quality of life reported by SCI patients, surpassing bladder dysfunction, problematic spasticity, pressure sores, and sexual dysfunctions [126]. However, whether pain directly affects cognition is still debated. Post-SCI pain may indirectly lead to cognitive decline and poor recovery by promoting immobility and sleep disturbance. Lack of physical activity is known to be a risk factor for cognitive impairment and dementia whereas moderate to high level of physical activities protect against cognitive impairments [127,128]. Given that most of SCI patients cannot participate in physical activity at an adequate level and only 50% of SCI patients engage in any physical activities during leisure time, it is not surprising that SCI patients are at higher risk of developing cognitive impairments [129,130].

In addition to the lower quality of life that patients experience due to pain and cognitive-emotional disturbance after SCI, depression can also impede the physical rehabilitation process and exacerbate health problems associated with SCI [15]. One study reported approximately one-third of SCI patients have symptoms of depression up to 10 years after injury while others found up to 78% of rehabilitation patients reported chronic pain [131,132]. Despite the difference in frequency, both studies consistently found SCI patients are more anxious and depressed than control subjects [133]. Avluk et al. reported a positive correlation between pain severity and the development of depression [134]. While the exact mechanisms remain to be seen, it is plausible that stress caused by persistent pain underlie these changes in neurological condition. Studies show a strong positive association between usual pain intensity and psychological distress, with significant differences in usual pain severity when those with and without possible clinical levels of anxiety and depression were compared [131,135]. This bidirectional relationship suggests that pain can lead to depression, and that depressed patients may be more sensitive to the pain. Indeed, a study combining rat models of chronic constriction injury and chronic mild stress showed that depressive-like behavior was associated with a heightened aversion to painful stimuli, implying depression can cause increased sensitivity to pain [136]. Those results suggest the possibility of a positive feedback loop wherein chronic SCI pain increases physiological distress and depression, which in turn increases sensitivity to pain. Together, with pain and depression impact cognitive function and recovery. A nationwide study conducted in Canada found that chronic SCI patients diagnosed with neuropathic pain and depression had increased risk of cardiovascular disease, such as heart disease and stroke [137]. Depression is also associated with a two-fold risk of dementia [138,139]. This may be partially due to reduced physical activities and leisure activities in depressed patients [140]. Thus, chronic pain not only increase the prevalence of depression in SCI patients, but depression may also lead to the development of dementia in SCI patients.

It is important to recognize that chronic pain is prevalent in SCI patients and significantly correlates with cognitive decline and depression. However, to what degree chronic pain causes those problems requires further investigation. The presence of unremitting pain may lead to dementia, cognitive dysfunction, and depression by altering patients’ behavior such as reducing physical activity and sleep quality. This differs from direct anterograde or retrograde signaling or altering physiological alternation such as inducing systemic inflammation; adding another layer complexity to how SCI induces cognitive and mood dysfunctions. Careful observation should be made to determine whether focused efforts on pain management in SCI patients can have extended benefits on cognitive and emotional well-being and vice versa.

## 7. Potential Therapeutic Intervention

### 7.1. Anti-Depressants

Due to the detrimental effects that mood disorders have on SCI patients, mood stabilization and maintaining good mental health is of the utmost importance to recovery and rehabilitation. As described above, depression is associated with decreased physical activity and increased risk of developing dementia [139,140]. Anti-depressant drugs can be advantageous in treating SCI-mediated depression, including improving mood status and quality of life following surgery, and reducing risk of delirium and suicide [141]. Venlafaxine may be more appropriate for patients with SCI presenting with depression and/or nociceptive pain [142,143,144]. In addition, antidepressant medications have been predicted to treat SCI pain. Some reports show an increased effectiveness of tricyclic antidepressants (TCA) on severe depression in SCI patients compared to those without SCI [145]. TCA may also be effective in ameliorating SCI-induced pain. An eight-week clinical trial showed that the TCA medication amitriptyline was significantly more effective at ameliorating neuropathic pain in SCI patients compared with diphenhydramine [146]. However, the efficacy of antidepressants in SCI pain management is still debated. The antidepressant duloxetine was reported to alleviate dynamic and cold allodynia but had no effect on tactile or pressure pain thresholds [147].

In experimental SCI models in rat, there is no correlation between locomotor functional recovery, assessed with the Basso, Beattie, and Bresnahan (BBB) scale, and performance on the depression tests including the sucrose preference, forced swim, open field, social exploration, and burrowing tasks [37], indicating the characterization of depression does not depend on motor recovery. One promising anti-depressant proven in experimental SCI is fluoxetine. The combined treatment of fluoxetine and treadmill gait training shows better BBB scale score in SCI rats than non-treated group or groups treated with fluoxetine or treadmill gait training alone [148]. Fluoxetine is a selective serotonin reuptake inhibitor that allows higher extracellular serotonin concentrations and prolonged activation of serotonin receptors. This might be one reason fluoxetine improve BBB score since serotonin receptor activation is known to contribute to motor function recovery after SCI [149]. Furthermore, fluoxetine is known to induce the production of neurotrophic factors and affect brain physiology. High dose fluoxetine (21 days of daily i.p. injections of 25 mg/kg) increased BDNF production and hippocampal neurogenesis in rats following SCI [150]. However, there were no synergistic effects of combining exercise and fluoxetine treatment on neurotrophic factor expression. Although BDNF concentrations did not change in the spinal cord with either physical activity or fluoxetine treatment, IGF-1 levels and cytogenesis were significantly decreased with high dose fluoxetine alone [150]. However, a low dose of fluoxetine (5 mg/kg/day) that has been shown to be subthreshold for increasing motor activity significantly decreased immobility in the forced swim test in depressed SCI rats without altering locomotor functional recovery [37]. These findings indicate fluoxetine has differential effects on the spinal cord and brain, including anti-inflammatory actions. Furthermore, whether improved mood by fluoxetine treatment affects cognitive function following SCI remains unclear. Ultimately, a better understanding of modifiable risk factors underlying cognitive impairment and depression in SCI subjects could lead to the development of more-effective interventions to treat these symptoms.

### 7.2. Cell Cycle Activation Inhibition

Although anti-depressants may potentially alleviate the symptoms of post-SCI depression, further research on the molecular mechanisms driving this neurological condition could yield more promising methods of treatment or prevention. One of these potential mechanisms involves cell cycle activation (CCA), which has been experimentally proven harmful after neurotrauma [151]. CCA is known an important secondary injury mechanism after TBI or SCI [57,60,152,153,154,155,156,157,158,159,160,161,162,163,164,165,166,167] that contributes to early posttraumatic neuronal cell death as well as to chronic neuroinflammation that leads to delayed progressive neurodegeneration. Using experimental SCI contusion models, we showed that treatment with cyclin-dependent kinase (CDK) inhibitors reduces neuronal death and microglial/astrocyte reactivity, attenuates lesion volume, and improves motor recovery [57,155,156,157,158,160]. Our recent data indicate that CCA increased not only in the lesioned spinal cord but also in various brain regions (thalamus, cortex, hippocampus) following SCI [38,39,57]. The expression of cell cycle genes are increased in multiple brain regions, where mRNA levels of cyclin A1, cyclin A2, cyclin D1, and PCNA genes were significantly increased as early as first few days after SCI and remained elevation at three months post-injury [38]. Cyclin D1 protein levels were also elevated in these regions [38,57]. Among initiating factors for CCA, the E2F1 transcription factor showed elevations in the hippocampus 24 h after injury in both rat and mouse, consistent with our previous report in the injured spinal cord [160]. E2F1-3 are members of the activator sub-family of E2F transcription factors and play a key upstream role in CCA [168,169]. Thus, E2Fs may represent a potential target to modulate these pathways. Importantly, early treatment with CR8, a potent inhibitor of multiple CDKs after injury largely prevented both the posttraumatic neuroinflammation and progressive neurodegeneration in the brain, as well as long-term cognitive dysfunction and depressive-like behavior [38,39]. As CCA activation causes cell death of post-mitotic cells (neurons, oligodendroglia) as well as activation and proliferation of mitotic cells (microglia, astrocytes) with neuroinflammation and secondary neurotoxicity [161], the effectiveness of CCA inhibitors likely involves multiple cell types.

### 7.3. Targeting Inflammation

A possible mechanism underlying the high dementia risk among patients with SCI is posttraumatic neuroinflammation and associated neurodegeneration. As the major cellular component of the innate immune system in the CNS, microglia play a critical role in the response to CNS trauma. In response to injury, microglia can produce neuroprotective factors, clear cellular debris and orchestrate neurorestorative processes that are beneficial for neurological recovery. However, dysregulated microglia can also produce high levels of pro-inflammatory and cytotoxic mediators that hinder CNS recovery [170,171]. Chronic neuroinflammation often continues for months to years after CNS trauma [170,171,172,173,174,175,176,177,178,179,180,181], contributing to delayed, progressive neuronal cell loss and neurological dysfunction. Chronic brain neuroinflammation after SCI is associated with progressive neurodegeneration in the brain regions associated with cognitive impairment [40]. Based on recent experimental work, an alternate hypothesis is that SCI-induced neuroinflammation affects hyperpathic pain, depression, and cognition. Considerable data also suggest that chronic neurotoxic inflammation may be a critical pathogenic mechanism in neurodegenerative disorders including AD [93,94]. SCI-mediated neuropsychological abnormalities are not necessarily “reactive” symptoms, but may reflect specific pathobiological changes that can be targeted [38,39,40,75]. Recent randomized clinical trials reported that targeting inflammation improves mood and neuropathic pain following SCI [76,182].

We and others have shown the importance of phagocytic NADPH oxidases (NOX2) in microglial activation and correlated production of pro-inflammatory factors, along with chronic neuronal cell loss and associated neurological dysfunction, after TBI or SCI [175,183,184,185,186]. Depletion or inhibition of NOX2 reduce reactive oxygen species (ROS) production and alter microglia/macrophage polarization balance toward the anti-inflammatory phenotype after neurotrauma [187,188,189], leading to neuroprotection. In a mouse SCI model, the combined use of the nonspecific apocynin and the AMPA receptor inhibitor NBQX resulted in reduced lesion volume and increased preservation of white matter and a greater overall improvement to functional recovery out to 6 weeks post-injury [190]. Systemically acute inhibition of NOX2 by NOX2ds-tat (a peptide that specific inhibits NOX2) can result in long-term alterations in function and microglial activity after SCI [186]. We have recently reported [188] that constitutive depletion or systemically inhibition of NOX2 significantly reduced mechanical/thermal cutaneous hypersensitivity and motor dysfunction after moderate contusion SCI at T10 in male mice. Thus, NOX2 signaling may be one of the key mechanisms of posttraumatic neuroinflammation after brain or spinal cord insults that can be effectively targeted by NOX2 inhibitors or gene knockout in TBI or SCI. Whether or not NOX2 activation contributes to subsequent brain inflammation and neurodegeneration following SCI is intriguing for future investigation.

Furthermore, targeting CNS resident microglial populations have been pursued as potential therapeutics for various neurodegenerative diseases [191], including neurotrauma, but most such approaches have lacked specificity and/or modest effectiveness in decreasing adult microglia. Genetic deletion of CX3CR1, a microglial chemokine receptor, promotes recovery after SCI, but this receptor is also highly expressed by infiltrating macrophages [192,193]. Recently developed transgenic mice (CX3CR1^CreER/+^:R26^iDTR/+^) permit depletion of resident microglia in the CNS but not blood CX3CR1^+^ cells [194]. Additionally, microglia are dependent on colony-stimulating factor 1 receptor (CSF1R) signaling for survival in the healthy adult brain [195]. Administration of CSF1R antagonists results in the rapid and continued elimination of virtually all microglia from the CNS, without significant effects on peripheral macrophage populations [195]. Mice lacking microglia, using this approach, are healthy, viable, and show no deleterious effects. Elimination of microglia is highly beneficial in CNS disease models [196,197,198,199], suggesting that CSF1R antagonists can be effective therapeutically for various CNS disorders associated with neuroinflammation. Crucially, CSF1R antagonists are in clinical trials for various cancers, and thus, these findings may be more readily translatable.

## 8. Conclusions and Perspectives

Across studies in human and experimental models, SCI-induced cognitive and mood disorders are consistently observed. fMRI imaging of the spinal cord reveals a reorganization of cortical networks in the brain following SCI in humans, including impairments in distributed cortical/subcortical networks that are engaged in information processing functions [9,200]. Recent preclinical work has begun to elucidate the underlying mechanisms of cognitive impairments following SCI, such as CCL21 release, neuroinflammation, and cell cycle pathways. However, the full picture of the connection between SCI and brain circuit dysfunction is yet to be uncovered. For example, anterograde and retrograde transport mechanisms are suggested to cause brain circuit dysfunctions after SCI, but the exact mechanisms involved are not yet understood.

In summary, cognitive dysfunction after SCI may reflect a combination of biochemical, physiological and behavioral alternations induced by SCI. Although potential clinical confounding factors include concomitant brain injury, psychological abnormality, as well as cardiovascular and sleep disorders, growing evidence demonstrates that SCI induces functional changes in the brain. Thus, it is important to view SCI as a brain degenerative disease in addition to a traditional understanding of SCI as a traumatic event. Future SCI rehabilitation efforts should better emphasize and examine the potential cognitive changes and mood disorders following SCI. Similarly, preclinical studies should further address these important translational issues in addressing injury mechanisms and therapeutic approaches to SCI.

## References

[1] Abramson C.E., McBride K.E., Konnyu K.J., Elliott S.L., Team S.R. (2008). Sexual health outcome measures for individuals with a spinal cord injury: A systematic review. Spinal Cord.

[2] Persu C., Caun V., Dragomiriteanu I., Geavlete P. (2009). Urological management of the patient with traumatic spinal cord injury. J. Med. Life.

[3] Siddall P.J., McClelland J.M., Rutkowski S.B., Cousins M.J. (2003). A longitudinal study of the prevalence and characteristics of pain in the first 5 years following spinal cord injury. Pain.

[4] Stormer S., Gerner H.J., Gruninger W., Metzmacher K., Follinger S., Wienke C., Aldinger W., Walker N., Zimmermann M., Paeslack V. (1997). Chronic pain/dysaesthesiae in spinal cord injury patients: Results of a multicentre study. Spinal Cord.

[5] Widerstrom-Noga E.G., Felix E.R., Cruz-Almeida Y., Turk D.C. (2007). Psychosocial subgroups in persons with spinal cord injuries and chronic pain. Arch. Phys. Med. Rehabil..

[6] Arango-Lasprilla J.C., Ketchum J.M., Starkweather A., Nicholls E., Wilk A.R. (2011). Factors predicting depression among persons with spinal cord injury 1 to 5 years post injury. NeuroRehabilitation.

[7] Davidoff G.N., Roth E.J., Richards J.S. (1992). Cognitive deficits in spinal cord injury: Epidemiology and outcome. Arch. Phys. Med. Rehabil..

[8] Dowler R.N., Harrington D.L., Haaland K.Y., Swanda R.M., Fee F., Fiedler K. (1997). Profiles of cognitive functioning in chronic spinal cord injury and the role of moderating variables. J. Int. Neuropsychol. Soc. Jins.

[9] Lazzaro I., Tran Y., Wijesuriya N., Craig A. (2013). Central correlates of impaired information processing in people with spinal cord injury. J. Clin. Neurophysiol. Off. Publ. Am. Electroencephalogr. Soc..

[10] Richards J.S., Brown L., Hagglund K., Bua G., Reeder K. (1988). Spinal cord injury and concomitant traumatic brain injury. Results of a longitudinal investigation. Am. J. Phys. Med. Rehabil..

[11] Roth E., Davidoff G., Thomas P., Doljanac R., Dijkers M., Berent S., Morris J., Yarkony G. (1989). A controlled study of neuropsychological deficits in acute spinal cord injury patients. Paraplegia.

[12] Umlauf R.L. (1992). Psychological interventions for chronic pain following spinal cord injury. Clin. J. Pain.

[13] Sachdeva R., Gao F., Chan C.C.H., Krassioukov A.V. (2018). Cognitive function after spinal cord injury: A systematic review. Neurology.

[14] Huang S.W., Wang W.T., Chou L.C., Liou T.H., Lin H.W. (2016). Risk of Dementia in Patients with Spinal Cord Injury: A Nationwide Population-Based Cohort Study. J. Neurotrauma.

[15] Murray R.F., Asghari A., Egorov D.D., Rutkowski S.B., Siddall P.J., Soden R.J., Ruff R. (2007). Impact of spinal cord injury on self-perceived pre- and postmorbid cognitive, emotional and physical functioning. Spinal Cord.

[16] Craig A., Guest R., Tran Y., Middleton J. (2017). Cognitive Impairment and Mood States after Spinal Cord Injury. J. Neurotrauma.

[17] Davidoff G., Thomas P., Johnson M., Berent S., Dijkers M., Doljanac R. (1988). Closed head injury in acute traumatic spinal cord injury: Incidence and risk factors. Arch. Phys. Med. Rehabil..

[18] Sauri J., Chamarro A., Gilabert A., Gifre M., Rodriguez N., Lopez-Blazquez R., Curcoll L., Benito-Penalva J., Soler D. (2017). Depression in Individuals With Traumatic and Nontraumatic Spinal Cord Injury Living in the Community. Arch. Phys. Med. Rehabil..

[19] Post M.W., van Leeuwen C.M. (2012). Psychosocial issues in spinal cord injury: A review. Spinal Cord.

[20] Craig A., Nicholson Perry K., Guest R., Tran Y., Dezarnaulds A., Hales A., Ephraums C., Middleton J. (2015). Prospective study of the occurrence of psychological disorders and comorbidities after spinal cord injury. Arch. Phys. Med. Rehabil..

[21] Migliorini C.E., New P.W., Tonge B.J. (2009). Comparison of depression, anxiety and stress in persons with traumatic and non-traumatic post-acute spinal cord injury. Spinal Cord.

[22] Shin J.C., Goo H.R., Yu S.J., Kim D.H., Yoon S.Y. (2012). Depression and Quality of Life in Patients within the First 6 Months after the Spinal Cord Injury. Ann. Rehabil Med..

[23] Barbonetti A., Cavallo F., D’Andrea S., Muselli M., Felzani G., Francavilla S., Francavilla F. (2017). Lower Vitamin D Levels Are Associated With Depression in People With Chronic Spinal Cord Injury. Arch. Phys. Med. Rehabil..

[24] Migliorini C., Sinclair A., Brown D., Tonge B., New P. (2015). Prevalence of mood disturbance in Australian adults with chronic spinal cord injury. Intern. Med. J..

[25] Migliorini C., Sinclair A., Brown D., Tonge B., New P. (2016). A randomised control trial of an Internet-based cognitive behaviour treatment for mood disorder in adults with chronic spinal cord injury. Spinal Cord.

[26] Endo T., Spenger C., Tominaga T., Brene S., Olson L. (2007). Cortical sensory map rearrangement after spinal cord injury: FMRI responses linked to Nogo signalling. Brain.

[27] Freund P., Weiskopf N., Ward N.S., Hutton C., Gall A., Ciccarelli O., Craggs M., Friston K., Thompson A.J. (2011). Disability, atrophy and cortical reorganization following spinal cord injury. Brain.

[28] Jurkiewicz M.T., Crawley A.P., Verrier M.C., Fehlings M.G., Mikulis D.J. (2006). Somatosensory cortical atrophy after spinal cord injury: A voxel-based morphometry study. Neurology.

[29] Wrigley P.J., Gustin S.M., Macey P.M., Nash P.G., Gandevia S.C., Macefield V.G., Siddall P.J., Henderson L.A. (2009). Anatomical changes in human motor cortex and motor pathways following complete thoracic spinal cord injury. Cereb. Cortex.

[30] Freund P., Wheeler-Kingshott C.A., Nagy Z., Gorgoraptis N., Weiskopf N., Friston K., Thompson A.J., Hutton C. (2012). Axonal integrity predicts cortical reorganisation following cervical injury. J. Neurol Neurosurg Psychiatry.

[31] Freund P., Weiskopf N., Ashburner J., Wolf K., Sutter R., Altmann D.R., Friston K., Thompson A., Curt A. (2013). MRI investigation of the sensorimotor cortex and the corticospinal tract after acute spinal cord injury: A prospective longitudinal study. Lancet Neurol.

[32] Nicotra A., Critchley H.D., Mathias C.J., Dolan R.J. (2006). Emotional and autonomic consequences of spinal cord injury explored using functional brain imaging. Brain.

[33] Ziegler G., Grabher P., Thompson A., Altmann D., Hupp M., Ashburner J., Friston K., Weiskopf N., Curt A., Freund P. (2018). Progressive neurodegeneration following spinal cord injury: Implications for clinical trials. Neurology.

[34] Seif M., Curt A., Thompson A.J., Grabher P., Weiskopf N., Freund P. (2018). Quantitative MRI of rostral spinal cord and brain regions is predictive of functional recovery in acute spinal cord injury. Neuroimage Clin..

[35] Seif M., Ziegler G., Freund P. (2018). Progressive Ventricles Enlargement and Cerebrospinal Fluid Volume Increases as a Marker of Neurodegeneration in Patients with Spinal Cord Injury: A Longitudinal Magnetic Resonance Imaging Study. J. Neurotrauma.

[36] Zhang B., Huang Y., Su Z., Wang S., Wang S., Wang J., Wang A., Lai X. (2011). Neurological, functional, and biomechanical characteristics after high-velocity behind armor blunt trauma of the spine. J. Trauma.

[37] Luedtke K., Bouchard S.M., Woller S.A., Funk M.K., Aceves M., Hook M.A. (2014). Assessment of depression in a rodent model of spinal cord injury. J. Neurotrauma.

[38] Wu J., Zhao Z., Sabirzhanov B., Stoica B.A., Kumar A., Luo T., Skovira J., Faden A.I. (2014). Spinal cord injury causes brain inflammation associated with cognitive and affective changes: Role of cell cycle pathways. J. Neurosci..

[39] Wu J., Stoica B.A., Luo T., Sabirzhanov B., Zhao Z., Guanciale K., Nayar S.K., Foss C.A., Pomper M.G., Faden A.I. (2014). Isolated spinal cord contusion in rats induces chronic brain neuroinflammation, neurodegeneration, and cognitive impairment: Involvement of cell cycle activation. Cell Cycle.

[40] Wu J., Zhao Z., Kumar A., Lipinski M.M., Loane D.J., Stoica B.A., Faden A.I. (2016). Endoplasmic Reticulum Stress and Disrupted Neurogenesis in the Brain Are Associated with Cognitive Impairment and Depressive-Like Behavior after Spinal Cord Injury. J. Neurotrauma.

[41] Popovich P.G., Horner P.J., Mullin B.B., Stokes B.T. (1996). A quantitative spatial analysis of the blood-spinal cord barrier. I. Permeability changes after experimental spinal contusion injury. Exp. Neurol..

[42] Noble L.J., Wrathall J.R. (1989). Distribution and time course of protein extravasation in the rat spinal cord after contusive injury. Brain Res..

[43] Whetstone W.D., Hsu J.Y., Eisenberg M., Werb Z., Noble-Haeusslein L.J. (2003). Blood-spinal cord barrier after spinal cord injury: Relation to revascularization and wound healing. J. Neurosci. Res..

[44] Cheng S., Gao W., Xu X., Fan H., Wu Y., Li F., Zhang J., Zhu X., Zhang Y. (2016). Methylprednisolone sodium succinate reduces BBB disruption and inflammation in a model mouse of intracranial haemorrhage. Brain Res. Bull..

[45] Kuriakose M., Rama Rao K.V., Younger D., Chandra N. (2018). Temporal and Spatial Effects of Blast Overpressure on Blood-Brain Barrier Permeability in Traumatic Brain Injury. Sci. Rep..

[46] Alonso-Calvino E., Martinez-Camero I., Fernandez-Lopez E., Humanes-Valera D., Foffani G., Aguilar J. (2016). Increased responses in the somatosensory thalamus immediately after spinal cord injury. Neurobiol Dis.

[47] Humanes-Valera D., Aguilar J., Foffani G. (2013). Reorganization of the intact somatosensory cortex immediately after spinal cord injury. PLoS ONE.

[48] Wang Q., Wang Z., Zhu P., Jiang J. (2004). Alterations of myelin basic protein and ultrastructure in the limbic system at the early stage of trauma-related stress disorder in dogs. J. Trauma.

[49] Lee B.H., Lee K.H., Kim U.J., Yoon D.H., Sohn J.H., Choi S.S., Yi I.G., Park Y.G. (2004). Injury in the spinal cord may produce cell death in the brain. Brain Res..

[50] Hains B.C., Black J.A., Waxman S.G. (2003). Primary cortical motor neurons undergo apoptosis after axotomizing spinal cord injury. J. Comp. Neurol..

[51] Chang C.M., Lee M.H., Wang T.C., Weng H.H., Chung C.Y., Yang J.T. (2009). Brain protection by methylprednisolone in rats with spinal cord injury. Neuroreport.

[52] Brock J.H., Rosenzweig E.S., Blesch A., Moseanko R., Havton L.A., Edgerton V.R., Tuszynski M.H. (2010). Local and remote growth factor effects after primate spinal cord injury. J. Neurosci..

[53] Nielson J.L., Strong M.K., Steward O. (2011). A reassessment of whether cortical motor neurons die following spinal cord injury. J. Comp. Neurol..

[54] Wannier T., Schmidlin E., Bloch J., Rouiller E.M. (2005). A unilateral section of the corticospinal tract at cervical level in primate does not lead to measurable cell loss in motor cortex. J. Neurotrauma.

[55] Zhao P., Waxman S.G., Hains B.C. (2007). Modulation of thalamic nociceptive processing after spinal cord injury through remote activation of thalamic microglia by cysteine cysteine chemokine ligand 21. J. Neurosci..

[56] Yoon E.J., Kim Y.K., Ik Shin H., Lee Y., Kim S.E. (2013). Cortical and white matter alterations in patients with neuropathic pain after spinal cord injury. Brain Res..

[57] Wu J., Raver C., Piao C., Keller A., Faden A.I. (2013). Cell cycle activation contributes to increased neuronal activity in the posterior thalamic nucleus and associated chronic hyperesthesia after rat spinal cord contusion. Neurotherapeutics.

[58] Wang G., Thompson S.M. (2008). Maladaptive homeostatic plasticity in a rodent model of central pain syndrome: Thalamic hyperexcitability after spinothalamic tract lesions. J. Neurosci..

[59] Masri R., Quiton R.L., Lucas J.M., Murray P.D., Thompson S.M., Keller A. (2009). Zona incerta: A role in central pain. J. Neurophysiol..

[60] Kabadi S.V., Stoica B.A., Loane D.J., Byrnes K.R., Hanscom M., Cabatbat R.M., Tan M.T., Faden A.I. (2012). Cyclin D1 gene ablation confers neuroprotection in traumatic brain injury. J. Neurotrauma.

[61] Knerlich-Lukoschus F., Noack M., von der Ropp-Brenner B., Lucius R., Mehdorn H.M., Held-Feindt J. (2011). Spinal cord injuries induce changes in CB1 cannabinoid receptor and C-C chemokine expression in brain areas underlying circuitry of chronic pain conditions. J. Neurotrauma.

[62] Koiv L., Merisalu E., Zilmer K., Tomberg T., Kaasik A.E. (1997). Changes of sympatho-adrenal and hypothalamo-pituitary-adrenocortical system in patients with head injury. Acta Neurol. Scand..

[63] Masgrau R., Servitja J.M., Young K.W., Pardo R., Sarri E., Nahorski S.R., Picatoste F. (2001). Characterization of the metabotropic glutamate receptors mediating phospholipase C activation and calcium release in cerebellar granule cells: Calcium-dependence of the phospholipase C response. Eur. J. Neurosci..

[64] Mason K.A., Hunter N.R., Raju U., Ariga H., Husain A., Valdecanas D., Neal R., Ang K.K., Milas L. (2004). Flavopiridol increases therapeutic ratio of radiotherapy by preferentially enhancing tumor radioresponse. Int. J. Radiat. Oncol. Biol. Phys..

[65] Hubscher C.H., Johnson R.D. (2006). Chronic spinal cord injury induced changes in the responses of thalamic neurons. Exp. Neurol..

[66] Hains B.C., Saab C.Y., Waxman S.G. (2005). Changes in electrophysiological properties and sodium channel Nav1.3 expression in thalamic neurons after spinal cord injury. Brain.

[67] Gwak Y.S., Kim H.K., Kim H.Y., Leem J.W. (2010). Bilateral hyperexcitability of thalamic VPL neurons following unilateral spinal injury in rats. J. Physiol. Sci. Jps.

[68] Gomez-Pinilla F., Ying Z., Zhuang Y. (2012). Brain and spinal cord interaction: Protective effects of exercise prior to spinal cord injury. PLoS ONE.

[69] Lau B.Y., Foldes A.E., Alieva N.O., Oliphint P.A., Busch D.J., Morgan J.R. (2011). Increased synapsin expression and neurite sprouting in lamprey brain after spinal cord injury. Exp. Neurol..

[70] Friberg H., Ferrand-Drake M., Bengtsson F., Halestrap A.P., Wieloch T. (1998). Cyclosporin A, but not FK 506, protects mitochondria and neurons against hypoglycemic damage and implicates the mitochondrial permeability transition in cell death. J. Neurosci..

[71] Fumagalli F., Madaschi L., Caffino L., Marfia G., Di Giulio A.M., Racagni G., Gorio A. (2009). Acute spinal cord injury reduces brain derived neurotrohic factor expression in rat hippocampus. Neuroscience.

[72] Wang H., Pullambhatla M., Guilarte T.R., Mease R.C., Pomper M.G. (2009). Synthesis of [(125)I]iodoDPA-713: A new probe for imaging inflammation. Biochem. Biophys. Res. Commun..

[73] Jure I., Pietranera L., De Nicola A.F., Labombarda F. (2017). Spinal Cord Injury Impairs Neurogenesis and Induces Glial Reactivity in the Hippocampus. Neurochem. Res..

[74] Xue W.K., Zhao W.J., Meng X.H., Shen H.F., Huang P.Z. (2019). Spinal cord injury induced Neuregulin 1 signaling changes in mouse prefrontal cortex and hippocampus. Brain Res. Bull..

[75] Maldonado-Bouchard S., Peters K., Woller S.A., Madahian B., Faghihi U., Patel S., Bake S., Hook M.A. (2016). Inflammation is increased with anxiety- and depression-like signs in a rat model of spinal cord injury. Brain Behav. Immun..

[76] Allison D.J., Ditor D.S. (2015). Targeting inflammation to influence mood following spinal cord injury: A randomized clinical trial. J. Neuroinflammation.

[77] Gage F.H. (2019). Adult neurogenesis in mammals. Science.

[78] Apple D.M., Fonseca R.S., Kokovay E. (2017). The role of adult neurogenesis in psychiatric and cognitive disorders. Brain Res..

[79] Zlomuzica A., Dere D., Binder S., De Souza Silva M.A., Huston J.P., Dere E. (2016). Neuronal histamine and cognitive symptoms in Alzheimer’s disease. Neuropharmacology.

[80] Couillard-Despres S. (2013). Hippocampal neurogenesis and ageing. Curr. Top. Behav. Neurosci..

[81] Henn F.A., Vollmayr B. (2004). Neurogenesis and depression: Etiology or epiphenomenon?. Biol. Psychiatry.

[82] Song C., Wang H. (2011). Cytokines mediated inflammation and decreased neurogenesis in animal models of depression. Prog. Neuro Psychopharmacol. Biol. Psychiatry.

[83] Sierra A., Beccari S., Diaz-Aparicio I., Encinas J.M., Comeau S., Tremblay M.E. (2014). Surveillance, phagocytosis, and inflammation: How never-resting microglia influence adult hippocampal neurogenesis. Neural Plast..

[84] Felix M.S., Popa N., Djelloul M., Boucraut J., Gauthier P., Bauer S., Matarazzo V.A. (2012). Alteration of forebrain neurogenesis after cervical spinal cord injury in the adult rat. Front. Neurosci..

[85] Franz S., Ciatipis M., Pfeifer K., Kierdorf B., Sandner B., Bogdahn U., Blesch A., Winner B., Weidner N. (2014). Thoracic rat spinal cord contusion injury induces remote spinal gliogenesis but not neurogenesis or gliogenesis in the brain. PLoS ONE.

[86] Boldrini M., Fulmore C.A., Tartt A.N., Simeon L.R., Pavlova I., Poposka V., Rosoklija G.B., Stankov A., Arango V., Dwork A.J. (2018). Human Hippocampal Neurogenesis Persists throughout Aging. Cell Stem Cell.

[87] Sorrells S.F., Paredes M.F., Cebrian-Silla A., Sandoval K., Qi D., Kelley K.W., James D., Mayer S., Chang J., Auguste K.I. (2018). Human hippocampal neurogenesis drops sharply in children to undetectable levels in adults. Nature.

[88] Kumar A., Stoica B.A., Sabirzhanov B., Burns M.P., Faden A.I., Loane D.J. (2013). Traumatic brain injury in aged animals increases lesion size and chronically alters microglial/macrophage classical and alternative activation states. Neurobiol Aging.

[89] von Leden R.E., Khayrullina G., Moritz K.E., Byrnes K.R. (2017). Age exacerbates microglial activation, oxidative stress, inflammatory and NOX2 gene expression, and delays functional recovery in a middle-aged rodent model of spinal cord injury. J. Neuroinflammation.

[90] Zhang B., Bailey W.M., McVicar A.L., Gensel J.C. (2016). Age increases reactive oxygen species production in macrophages and potentiates oxidative damage after spinal cord injury. Neurobiol. Aging.

[91] Fenn A.M., Hall J.C., Gensel J.C., Popovich P.G., Godbout J.P. (2014). IL-4 signaling drives a unique arginase+/IL-1beta+ microglia phenotype and recruits macrophages to the inflammatory CNS: Consequences of age-related deficits in IL-4Ralpha after traumatic spinal cord injury. J. Neurosci..

[92] Hooshmand M.J., Galvan M.D., Partida E., Anderson A.J. (2014). Characterization of recovery, repair, and inflammatory processes following contusion spinal cord injury in old female rats: Is age a limitation?. Immun. Ageing.

[93] Eikelenboom P., van Exel E., Hoozemans J.J., Veerhuis R., Rozemuller A.J., van Gool W.A. (2010). Neuroinflammation—An early event in both the history and pathogenesis of Alzheimer’s disease. Neurodegener Dis.

[94] Perry G.M., Sagvolden T., Faraone S.V. (2010). Intraindividual variability (IIV) in an animal model of ADHD—the Spontaneously Hypertensive Rat. Behav. Brain Funct..

[95] Graafmans W.C., Ooms M.E., Hofstee H.M., Bezemer P.D., Bouter L.M., Lips P. (1996). Falls in the elderly: A prospective study of risk factors and risk profiles. Am. J. Epidemiol.

[96] Tinetti M.E., Williams C.S. (1997). Falls, injuries due to falls, and the risk of admission to a nursing home. N. Engl. J. Med..

[97] Buchner D.M., Larson E.B. (1987). Falls and fractures in patients with Alzheimer-type dementia. JAMA.

[98] Allan L.M., Ballard C.G., Rowan E.N., Kenny R.A. (2009). Incidence and prediction of falls in dementia: A prospective study in older people. PLoS ONE.

[99] van Doorn C., Gruber-Baldini A.L., Zimmerman S., Hebel J.R., Port C.L., Baumgarten M., Quinn C.C., Taler G., May C., Magaziner J. (2003). Dementia as a risk factor for falls and fall injuries among nursing home residents. J. Am. Geriatr. Soc..

[100] de Jong E.K., Vinet J., Stanulovic V.S., Meijer M., Wesseling E., Sjollema K., Boddeke H.W., Biber K. (2008). Expression, transport, and axonal sorting of neuronal CCL21 in large dense-core vesicles. Faseb J..

[101] de Jong E.K., Dijkstra I.M., Hensens M., Brouwer N., van Amerongen M., Liem R.S., Boddeke H.W., Biber K. (2005). Vesicle-mediated transport and release of CCL21 in endangered neurons: A possible explanation for microglia activation remote from a primary lesion. J. Neurosci..

[102] Biber K., Tsuda M., Tozaki-Saitoh H., Tsukamoto K., Toyomitsu E., Masuda T., Boddeke H., Inoue K. (2011). Neuronal CCL21 up-regulates microglia P2X4 expression and initiates neuropathic pain development. Embo J..

[103] Biber K., Sauter A., Brouwer N., Copray S.C., Boddeke H.W. (2001). Ischemia-induced neuronal expression of the microglia attracting chemokine Secondary Lymphoid-tissue Chemokine (SLC). Glia.

[104] de Haas A.H., van Weering H.R., de Jong E.K., Boddeke H.W., Biber K.P. (2007). Neuronal chemokines: Versatile messengers in central nervous system cell interaction. Mol. Neurobiol..

[105] Biber K., Boddeke E. (2014). Neuronal CC chemokines: The distinct roles of CCL21 and CCL2 in neuropathic pain. Front. Cell. Neurosci..

[106] Old E.A., Malcangio M. (2012). Chemokine mediated neuron-glia communication and aberrant signalling in neuropathic pain states. Curr. Opin. Pharmacol..

[107] Banati R.B. (2002). Brain plasticity and microglia: Is transsynaptic glial activation in the thalamus after limb denervation linked to cortical plasticity and central sensitisation?. J. Physiol. Paris.

[108] Gerard C., Gerard N.P. (2001). Chemokines: Back to the future?. Nat. Cell Biol.

[109] Kumar A., Stoica B.A., Loane D.J., Yang M., Abulwerdi G., Khan N., Kumar A., Thom S.R., Faden A.I. (2017). Microglial-derived microparticles mediate neuroinflammation after traumatic brain injury. J. Neuroinflammation.

[110] Guan Z., Kuhn J.A., Wang X., Colquitt B., Solorzano C., Vaman S., Guan A.K., Evans-Reinsch Z., Braz J., Devor M. (2016). Injured sensory neuron-derived CSF1 induces microglial proliferation and DAP12-dependent pain. Nat. Neurosci..

[111] van Weering H.R., de Jong A.P., de Haas A.H., Biber K.P., Boddeke H.W. (2010). CCL21-induced calcium transients and proliferation in primary mouse astrocytes: CXCR3-dependent and independent responses. Brain Behav. Immun..

[112] Biber K., de Jong E.K., van Weering H.R., Boddeke H.W. (2006). Chemokines and their receptors in central nervous system disease. Curr. Drug Targets.

[113] Biber K., Zuurman M.W., Dijkstra I.M., Boddeke H.W. (2002). Chemokines in the brain: Neuroimmunology and beyond. Curr. Opin. Pharmacol..

[114] Rappert A., Biber K., Nolte C., Lipp M., Schubel A., Lu B., Gerard N.P., Gerard C., Boddeke H.W., Kettenmann H. (2002). Secondary lymphoid tissue chemokine (CCL21) activates CXCR3 to trigger a Cl- current and chemotaxis in murine microglia. J. Immunol..

[115] Dijkstra I.M., de Haas A.H., Brouwer N., Boddeke H.W., Biber K. (2006). Challenge with innate and protein antigens induces CCR7 expression by microglia in vitro and in vivo. Glia.

[116] Sun X., Jones Z.B., Chen X.M., Zhou L., So K.F., Ren Y. (2016). Multiple organ dysfunction and systemic inflammation after spinal cord injury: A complex relationship. J. Neuroinflammation.

[117] Bao F., Bailey C.S., Gurr K.R., Bailey S.I., Rosas-Arellano M.P., Dekaban G.A., Weaver L.C. (2009). Increased oxidative activity in human blood neutrophils and monocytes after spinal cord injury. Exp. Neurol..

[118] Bigford G.E., Bracchi-Ricard V.C., Keane R.W., Nash M.S., Bethea J.R. (2013). Neuroendocrine and cardiac metabolic dysfunction and NLRP3 inflammasome activation in adipose tissue and pancreas following chronic spinal cord injury in the mouse. ASN Neuro.

[119] Hasturk A., Atalay B., Calisaneller T., Ozdemir O., Oruckaptan H., Altinors N. (2009). Analysis of serum pro-inflammatory cytokine levels after rat spinal cord ischemia/reperfusion injury and correlation with tissue damage. Turk. Neurosurg.

[120] Lerch J.K., Puga D.A., Bloom O., Popovich P.G. (2014). Glucocorticoids and macrophage migration inhibitory factor (MIF) are neuroendocrine modulators of inflammation and neuropathic pain after spinal cord injury. Semin. Immunol..

[121] Ankeny D.P., Popovich P.G. (2009). Mechanisms and implications of adaptive immune responses after traumatic spinal cord injury. Neuroscience.

[122] Lucin K.M., Sanders V.M., Jones T.B., Malarkey W.B., Popovich P.G. (2007). Impaired antibody synthesis after spinal cord injury is level dependent and is due to sympathetic nervous system dysregulation. Exp. Neurol..

[123] Popovich P., McTigue D. (2009). Damage control in the nervous system: Beware the immune system in spinal cord injury. Nat. Med..

[124] Burke D., Fullen B.M., Stokes D., Lennon O. (2017). Neuropathic pain prevalence following spinal cord injury: A systematic review and meta-analysis. Eur. J. Pain.

[125] Nicholson Perry K., Nicholas M.K., Middleton J. (2009). Spinal cord injury-related pain in rehabilitation: A cross-sectional study of relationships with cognitions, mood and physical function. Eur. J. Pain.

[126] Westgren N., Levi R. (1998). Quality of life and traumatic spinal cord injury. Arch. Phys. Med. Rehabil..

[127] Kirk-Sanchez N.J., McGough E.L. (2014). Physical exercise and cognitive performance in the elderly: Current perspectives. Clin. Interv. Aging.

[128] Sofi F., Valecchi D., Bacci D., Abbate R., Gensini G.F., Casini A., Macchi C. (2011). Physical activity and risk of cognitive decline: A meta-analysis of prospective studies. J. Intern. Med..

[129] Ginis K.A., Hicks A.L., Latimer A.E., Warburton D.E., Bourne C., Ditor D.S., Goodwin D.L., Hayes K.C., McCartney N., McIlraith A. (2011). The development of evidence-informed physical activity guidelines for adults with spinal cord injury. Spinal Cord.

[130] Ginis K.A., Latimer A.E., Arbour-Nicitopoulos K.P., Buchholz A.C., Bray S.R., Craven B.C., Hayes K.C., Hicks A.L., McColl M.A., Potter P.J. (2010). Leisure time physical activity in a population-based sample of people with spinal cord injury part I: Demographic and injury-related correlates. Arch. Phys. Med. Rehabil..

[131] Pollard C., Kennedy P. (2007). A longitudinal analysis of emotional impact, coping strategies and post-traumatic psychological growth following spinal cord injury: A 10-year review. Br. J. Health Psychol..

[132] Ataoglu E., Tiftik T., Kara M., Tunc H., Ersoz M., Akkus S. (2013). Effects of chronic pain on quality of life and depression in patients with spinal cord injury. Spinal Cord.

[133] Hancock K.M., Craig A.R., Dickson H.G., Chang E., Martin J. (1993). Anxiety and depression over the first year of spinal cord injury: A longitudinal study. Paraplegia.

[134] Avluk O.C., Gurcay E., Gurcay A.G., Karaahmet O.Z., Tamkan U., Cakci A. (2014). Effects of chronic pain on function, depression, and sleep among patients with traumatic spinal cord injury. Ann. Saudi Med..

[135] Kennedy P., Kilvert A., Hasson L. (2016). A 21-year longitudinal analysis of impact, coping, and appraisals following spinal cord injury. Rehabil. Psychol..

[136] Bravo L., Mico J.A., Rey-Brea R., Perez-Nievas B., Leza J.C., Berrocoso E. (2012). Depressive-like states heighten the aversion to painful stimuli in a rat model of comorbid chronic pain and depression. Anesthesiology.

[137] Cragg J.J., Noonan V.K., Noreau L., Borisoff J.F., Kramer J.K. (2015). Neuropathic pain, depression, and cardiovascular disease: A national multicenter study. Neuroepidemiology.

[138] Diniz B.S., Butters M.A., Albert S.M., Dew M.A., Reynolds C.F. (2013). Late-life depression and risk of vascular dementia and Alzheimer’s disease: Systematic review and meta-analysis of community-based cohort studies. Br. J. Psychiatry J. Ment. Sci..

[139] Ownby R.L., Crocco E., Acevedo A., John V., Loewenstein D. (2006). Depression and risk for Alzheimer disease: Systematic review, meta-analysis, and metaregression analysis. Arch. Gen. Psychiatry.

[140] Mulroy S.J., Hatchett P.E., Eberly V.J., Haubert L.L., Conners S., Gronley J., Garshick E., Requejo P.S. (2016). Objective and Self-Reported Physical Activity Measures and Their Association With Depression and Satisfaction With Life in Persons With Spinal Cord Injury. Arch. Phys. Med. Rehabil..

[141] Bayoumi A.B., Ikizgul O., Karaali C.N., Bozkurt S., Konya D., Toktas Z.O. (2019). Antidepressants in Spine Surgery: A Systematic Review to Determine Benefits and Risks. Asian Spine J..

[142] Fann J.R., Bombardier C.H., Richards J.S., Wilson C.S., Heinemann A.W., Warren A.M., Brooks L., McCullumsmith C.B., Temkin N.R., Warms C. (2015). Venlafaxine extended-release for depression following spinal cord injury: A randomized clinical trial. Jama Psychiatry.

[143] Richards J.S., Bombardier C.H., Wilson C.S., Chiodo A.E., Brooks L., Tate D.G., Temkin N.R., Barber J.K., Heinemann A.W., McCullumsmith C. (2015). Efficacy of venlafaxine XR for the treatment of pain in patients with spinal cord injury and major depression: A randomized, controlled trial. Arch. Phys. Med. Rehabil..

[144] Tate D.G., Forchheimer M., Bombardier C.H., Heinemann A.W., Neumann H.D., Fann J.R. (2015). Differences in quality of life outcomes among depressed spinal cord injury trial participants. Arch. Phys. Med. Rehabil..

[145] Kim S.P., Davis S.W., Sell G.H. (1977). Amitriptyline in severely depressed spinal cord-injured patients: Rapidity of response. Arch. Phys. Med. Rehabil..

[146] Rintala D.H., Holmes S.A., Courtade D., Fiess R.N., Tastard L.V., Loubser P.G. (2007). Comparison of the effectiveness of amitriptyline and gabapentin on chronic neuropathic pain in persons with spinal cord injury. Arch. Phys. Med. Rehabil..

[147] Vranken J.H., Hollmann M.W., van der Vegt M.H., Kruis M.R., Heesen M., Vos K., Pijl A.J., Dijkgraaf M.G. (2011). Duloxetine in patients with central neuropathic pain caused by spinal cord injury or stroke: A randomized, double-blind, placebo-controlled trial. Pain.

[148] Cristante A.F., Filho T.E., Oliveira R.P., Marcon R.M., Ferreira R., Santos G.B. (2013). Effects of antidepressant and treadmill gait training on recovery from spinal cord injury in rats. Spinal Cord.

[149] Murray K.C., Nakae A., Stephens M.J., Rank M., D’Amico J., Harvey P.J., Li X., Harris R.L., Ballou E.W., Anelli R. (2010). Recovery of motoneuron and locomotor function after spinal cord injury depends on constitutive activity in 5-HT2C receptors. Nat. Med..

[150] Engesser-Cesar C., Anderson A.J., Cotman C.W. (2007). Wheel running and fluoxetine antidepressant treatment have differential effects in the hippocampus and the spinal cord. Neuroscience.

[151] Wu J.F., Stoica B.A., Faden A.I. (2011). Cell Cycle Activation and Spinal Cord Injury. Neurotherapeutics.

[152] Tian D.S., Dong Q., Pan D.J., He Y., Yu Z.Y., Xie M.J., Wang W. (2007). Attenuation of astrogliosis by suppressing of microglial proliferation with the cell cycle inhibitor olomoucine in rat spinal cord injury model. Brain Res..

[153] Tian D.S., Yu Z.Y., Xie M.J., Bu B.T., Witte O.W., Wang W. (2006). Suppression of astroglial scar formation and enhanced axonal regeneration associated with functional recovery in a spinal cord injury rat model by the cell cycle inhibitor olomoucine. J. Neurosci. Res..

[154] Tian D.S., Xie M.J., Yu Z.Y., Zhang Q., Wang Y.H., Chen B., Chen C., Wang W. (2007). Cell cycle inhibition attenuates microglia induced inflammatory response and alleviates neuronal cell death after spinal cord injury in rats. Brain Res..

[155] Wu J., Stoica B.A., Dinizo M., Pajoohesh-Ganji A., Piao C., Faden A.I. (2012). Delayed cell cycle pathway modulation facilitates recovery after spinal cord injury. Cell Cycle.

[156] Wu J., Renn C.L., Faden A.I., Dorsey S.G. (2013). TrkB.T1 contributes to neuropathic pain after spinal cord injury through regulation of cell cycle pathways. J. Neurosci..

[157] Wu J., Pajoohesh-Ganji A., Stoica B.A., Dinizo M., Guanciale K., Faden A.I. (2012). Delayed expression of cell cycle proteins contributes to astroglial scar formation and chronic inflammation after rat spinal cord contusion. J. Neuroinflammation.

[158] Byrnes K.R., Stoica B.A., Fricke S., Di Giovanni S., Faden A.I. (2007). Cell cycle activation contributes to post-mitotic cell death and secondary damage after spinal cord injury. Brain.

[159] Di Giovanni S., Knoblach S.M., Brandoli C., Aden S.A., Hoffman E.P., Faden A.I. (2003). Gene profiling in spinal cord injury shows role of cell cycle in neuronal death. Ann. Neurol.

[160] Wu J., Kharebava G., Piao C., Stoica B.A., Dinizo M., Sabirzhanov B., Hanscom M., Guanciale K., Faden A.I. (2012). Inhibition of E2F1/CDK1 pathway attenuates neuronal apoptosis in vitro and confers neuroprotection after spinal cord injury in vivo. PLoS ONE.

[161] Di Giovanni S., Movsesyan V., Ahmed F., Cernak I., Schinelli S., Stoica B., Faden A.I. (2005). Cell cycle inhibition provides neuroprotection and reduces glial proliferation and scar formation after traumatic brain injury. Proc. Natl. Acad. Sci. USA.

[162] Cernak I., Stoica B., Byrnes K.R., Di Giovanni S., Faden A.I. (2005). Role of the cell cycle in the pathobiology of central nervous system trauma. Cell Cycle.

[163] Hilton G.D., Stoica B.A., Byrnes K.R., Faden A.I. (2008). Roscovitine reduces neuronal loss, glial activation, and neurologic deficits after brain trauma. J. Cereb Blood Flow Metab.

[164] Kabadi S.V., Stoica B.A., Byrnes K.R., Hanscom M., Loane D.J., Faden A.I. (2012). Selective CDK inhibitor limits neuroinflammation and progressive neurodegeneration after brain trauma. J. Cereb Blood Flow Metab.

[165] Kabadi S.V., Stoica B.A., Hanscom M., Loane D.J., Kharebava G., Murray Ii M.G., Cabatbat R.M., Faden A.I. (2012). CR8, a selective and potent CDK inhibitor, provides neuroprotection in experimental traumatic brain injury. Neurotherapeutics.

[166] Kabadi S.V., Stoica B.A., Loane D.J., Luo T., Faden A.I. (2014). CR8, a novel inhibitor of CDK, limits microglial activation, astrocytosis, neuronal loss, and neurologic dysfunction after experimental traumatic brain injury. J. Cereb Blood Flow Metab.

[167] Skovira J.W., Wu J., Matyas J.J., Kumar A., Hanscom M., Kabadi S.V., Fang R., Faden A.I. (2016). Cell cycle inhibition reduces inflammatory responses, neuronal loss, and cognitive deficits induced by hypobaria exposure following traumatic brain injury. J. Neuroinflammation.

[168] Greene L.A., Biswas S.C., Liu D.X. (2004). Cell cycle molecules and vertebrate neuron death: E2F at the hub. Cell Death Differ..

[169] Nahle Z., Polakoff J., Davuluri R.V., McCurrach M.E., Jacobson M.D., Narita M., Zhang M.Q., Lazebnik Y., Bar-Sagi D., Lowe S.W. (2002). Direct coupling of the cell cycle and cell death machinery by E2F. Nat. Cell Biol..

[170] Hesp Z.C., Goldstein E.Z., Miranda C.J., Kaspar B.K., McTigue D.M. (2015). Chronic oligodendrogenesis and remyelination after spinal cord injury in mice and rats. J. Neurosci..

[171] Plemel J.R., Wee Yong V., Stirling D.P. (2014). Immune modulatory therapies for spinal cord injury--past, present and future. Exp. Neurol..

[172] Acosta S.A., Tajiri N., Shinozuka K., Ishikawa H., Grimmig B., Diamond D., Sanberg P.R., Bickford P.C., Kaneko Y., Borlongan C.V. (2013). Long-term upregulation of inflammation and suppression of cell proliferation in the brain of adult rats exposed to traumatic brain injury using the controlled cortical impact model. PLoS ONE.

[173] Aungst S.L., Kabadi S.V., Thompson S.M., Stoica B.A., Faden A.I. (2014). Repeated mild traumatic brain injury causes chronic neuroinflammation, changes in hippocampal synaptic plasticity, and associated cognitive deficits. J. Cereb Blood Flow Metab.

[174] Johnson V.E., Stewart J.E., Begbie F.D., Trojanowski J.Q., Smith D.H., Stewart W. (2013). Inflammation and white matter degeneration persist for years after a single traumatic brain injury. Brain.

[175] Loane D.J., Kumar A., Stoica B.A., Cabatbat R., Faden A.I. (2014). Progressive neurodegeneration after experimental brain trauma: Association with chronic microglial activation. J. Neuropathol. Exp. Neurol..

[176] Mouzon B.C., Bachmeier C., Ferro A., Ojo J.O., Crynen G., Acker C.M., Davies P., Mullan M., Stewart W., Crawford F. (2014). Chronic neuropathological and neurobehavioral changes in a repetitive mild traumatic brain injury model. Ann. Neurol..

[177] Nagamoto-Combs K., McNeal D.W., Morecraft R.J., Combs C.K. (2007). Prolonged microgliosis in the rhesus monkey central nervous system after traumatic brain injury. J. Neurotrauma.

[178] Nonaka M., Chen X.H., Pierce J.E., Leoni M.J., McIntosh T.K., Wolf J.A., Smith D.H. (1999). Prolonged activation of NF-kappaB following traumatic brain injury in rats. J. Neurotrauma.

[179] Ramlackhansingh A.F., Brooks D.J., Greenwood R.J., Bose S.K., Turkheimer F.E., Kinnunen K.M., Gentleman S., Heckemann R.A., Gunanayagam K., Gelosa G. (2011). Inflammation after trauma: Microglial activation and traumatic brain injury. Ann. Neurol..

[180] Smith C., Gentleman S.M., Leclercq P.D., Murray L.S., Griffin W.S., Graham D.I., Nicoll J.A. (2013). The neuroinflammatory response in humans after traumatic brain injury. Neuropathol. Appl. Neurobiol..

[181] Huang S.W., Wang W.T., Chou L.C., Liou T.H., Chen Y.W., Lin H.W. (2016). Diabetes mellitus increases the risk of rotator cuff tear repair surgery: A population-based cohort study. J. Diabetes Its Complicat..

[182] Allison D.J., Thomas A., Beaudry K., Ditor D.S. (2016). Targeting inflammation as a treatment modality for neuropathic pain in spinal cord injury: A randomized clinical trial. J. Neuroinflammation.

[183] Loane D.J., Stoica B.A., Pajoohesh-Ganji A., Byrnes K.R., Faden A.I. (2009). Activation of metabotropic glutamate receptor 5 modulates microglial reactivity and neurotoxicity by inhibiting NADPH oxidase. J. Biol Chem.

[184] Loane D.J., Stoica B.A., Byrnes K.R., Jeong W., Faden A.I. (2013). Activation of mGluR5 and inhibition of NADPH oxidase improves functional recovery after traumatic brain injury. J. Neurotrauma.

[185] Cooney S.J., Zhao Y., Byrnes K.R. (2014). Characterization of the expression and inflammatory activity of NADPH oxidase after spinal cord injury. Free Radic. Res..

[186] Khayrullina G., Bermudez S., Byrnes K.R. (2015). Inhibition of NOX2 reduces locomotor impairment, inflammation, and oxidative stress after spinal cord injury. J. Neuroinflammation.

[187] von Leden R.E., Yauger Y.J., Khayrullina G., Byrnes K.R. (2016). Central Nervous System Injury and Nicotinamide Adenine Dinucleotide Phosphate Oxidase: Oxidative Stress and Therapeutic Targets. J. Neurotrauma.

[188] Sabirzhanov B., Li Y., Coll-Miro M., Matyas J.J., He J., Kumar A., Ward N., Yu J., Faden A.I., Wu J. (2019). Inhibition of NOX2 signaling limits pain-related behavior and improves motor function in male mice after spinal cord injury: Participation of IL-10/miR-155 pathways. Brain Behav. Immun..

[189] Barrett J.P., Henry R.J., Villapol S., Stoica B.A., Kumar A., Burns M.P., Faden A.I., Loane D.J. (2017). NOX2 deficiency alters macrophage phenotype through an IL-10/STAT3 dependent mechanism: Implications for traumatic brain injury. J. Neuroinflammation.

[190] Johnstone J.T., Morton P.D., Jayakumar A.R., Johnstone A.L., Gao H., Bracchi-Ricard V., Pearse D.D., Norenberg M.D., Bethea J.R. (2013). Inhibition of NADPH oxidase activation in oligodendrocytes reduces cytotoxicity following trauma. PLoS ONE.

[191] Subramaniam S.R., Federoff H.J. (2017). Targeting Microglial Activation States as a Therapeutic Avenue in Parkinson’s Disease. Front. Aging Neurosci..

[192] Donnelly D.J., Longbrake E.E., Shawler T.M., Kigerl K.A., Lai W., Tovar C.A., Ransohoff R.M., Popovich P.G. (2011). Deficient CX3CR1 signaling promotes recovery after mouse spinal cord injury by limiting the recruitment and activation of Ly6Clo/iNOS+ macrophages. J. Neurosci..

[193] Freria C.M., Hall J.C., Wei P., Guan Z., McTigue D.M., Popovich P.G. (2017). Deletion of the Fractalkine Receptor, CX3CR1, Improves Endogenous Repair, Axon Sprouting, and Synaptogenesis after Spinal Cord Injury in Mice. J. Neurosci..

[194] Peng J., Gu N., Zhou L., U B.E., Murugan M., Gan W.B., Wu L.J. (2016). Microglia and monocytes synergistically promote the transition from acute to chronic pain after nerve injury. Nat. Commun..

[195] Elmore M.R., Najafi A.R., Koike M.A., Dagher N.N., Spangenberg E.E., Rice R.A., Kitazawa M., Matusow B., Nguyen H., West B.L. (2014). Colony-stimulating factor 1 receptor signaling is necessary for microglia viability, unmasking a microglia progenitor cell in the adult brain. Neuron.

[196] Rice R.A., Spangenberg E.E., Yamate-Morgan H., Lee R.J., Arora R.P., Hernandez M.X., Tenner A.J., West B.L., Green K.N. (2015). Elimination of Microglia Improves Functional Outcomes Following Extensive Neuronal Loss in the Hippocampus. J. Neurosci..

[197] Dagher N.N., Najafi A.R., Kayala K.M., Elmore M.R., White T.E., Medeiros R., West B.L., Green K.N. (2015). Colony-stimulating factor 1 receptor inhibition prevents microglial plaque association and improves cognition in 3xTg-AD mice. J. Neuroinflammation.

[198] Acharya M.M., Green K.N., Allen B.D., Najafi A.R., Syage A., Minasyan H., Le M.T., Kawashita T., Giedzinski E., Parihar V.K. (2016). Elimination of microglia improves cognitive function following cranial irradiation. Sci. Rep..

[199] Walter T.J., Crews F.T. (2017). Microglial depletion alters the brain neuroimmune response to acute binge ethanol withdrawal. J. Neuroinflammation.

[200] Lotze M., Laubis-Herrmann U., Topka H. (2006). Combination of TMS and fMRI reveals a specific pattern of reorganization in M1 in patients after complete spinal cord injury. Restor. Neurol. Neurosci..

